# A seabird’s eye view: visual fields of some seabirds (Laridae and Procellariidae) from tropical latitudes

**DOI:** 10.1007/s00114-024-01926-4

**Published:** 2024-07-17

**Authors:** Eleanor A. Lucas, Graham R. Martin, Gérard Rocamora, Steven J. Portugal

**Affiliations:** 1 Department of Biological Sciences, School of Life and Environmental Sciences, Royal Holloway, University of London, Egham, Surrey TW20 0EX UK; 2 School of Biosciences, University of Birmingham, Edgbaston, Birmingham, B15 2TT UK; 3 Island Conservation Society, Mahé, Seychelles; 4 Island Biodiversity and Conservation Centre, University of Seychelles, Mahé, Seychelles; 5 The Natural History Museum Tring, Akeman Street, Tring, Herts HP23 6AP UK; 6 Department of Biology, University of Oxford, Oxford, OX1 3SZ UK

**Keywords:** Binocularity, Foraging ecology, Ophthalmoscope, Seychelles

## Abstract

The visual field of a bird defines the amount of information that can be extracted from the environment around it, using the eyes. Previous visual field research has left large phylogenetic gaps, where tropical bird species have been comparatively understudied. Using the ophthalmoscopic technique, we measured the visual fields of seven tropical seabird species, to understand what are the primary determinants of their visual fields. The visual field topographies of the seven seabird species were relatively similar, despite the two groups of Terns (Laridae) and Shearwaters (Procellariidae) being phylogenetically distant. We propose this similarity is due to their largely similar foraging ecology. These findings support previous research that foraging ecology rather than relatedness is the key determining factor behind a bird’s visual field topography. Some bird species were identified to have more limited binocular fields, such as Brown Noddies (*Anous stolidus*) where binocularity onsets lower down within the visual field, resulting in a larger blind area about the head.

**Supplementary Information:**

The online version contains supplementary material available at 10.1007/s00114-024-01926-4.

## Introduction

Differences in the optical structure, size, and movement of eyes influence the quality of images formed, and the extent of the world seen from moment to moment (Martin 2007, 2012). The visual field of an eye, and how the fields of the two eyes are combined, define the extent of the world from which an animal can extract information at any instant, and is regarded as a key component in describing the sensory ecology of an animal (Martin 1994). The visual field of an animal has three main components: the monocular field describes the extent of the world seen by each eye, the binocular field describes the region where the monocular fields overlap, and the blind areas describe the region in which no vision is provided (Waldvogel 1990). The cyclopean field describes the total region about the head from which information can be extracted at any one moment, by either monocular or binocular vision (Fernández-Juricic et al. 2004; Martin 1994).

Among birds, the topography of the binocular field (the size, shape, and position of the region of binocular overlap) shows considerable interspecific variation (Burton 2008; Martin 2017). These variations are dependent on the extent of the monocular field of each eye and the positioning of the eyes in the skull (Martin 2017). In general, eyes that are placed more laterally in the skull provide smaller binocular fields but larger cyclopean fields. It has been hypothesized that interspecific differences in binocular field topography among birds are primarily influenced by species-specific sensory requirements of foraging, rather than factors such as shared ancestry or the guidance of locomotion (Cantlay et al. 2019, 2023; Martin 1999; Potier et al. 2018).

Visual fields have been determined in over 180 bird species (Martin 2017), including 14 species of seabirds: two species of penguins (Spheniscidae), two species of Albatrosses (Diomedeidae), three species of petrels and shearwaters (Procellariidae), one species of cormorant (Phalacrocoracidae), and two species of auks (Alcidae). In this study, we determined the visual field in a further seven species of seabirds which inhabit tropical regions (between latitudes 23.5° north and south): five species of terns (Laridae), and two species of shearwaters (Procellariidae). These two taxa are distant from each other in the phylogenetic tree, but share a generally similar habitat and foraging technique, and we use data on their visual fields to test further the hypothesis that foraging technique, rather than phylogenetic relatedness, is the primary influence on visual field topography in birds (sensu Cantlay et al. 2023).

## Materials and methods

Visual fields were measured in seven species of tropical seabirds from two avian families, Laridae (Charadriiformes) and Procellariidae (Procellariiformes) (Table 1). All species are described as foraging by dipping or foraging by plunging (Tobias et al. 2022) (Supplementary Information Table S2). Dipping is where food items are taken from the surface of the water or just below while plunging (or plunge diving) is where the bird enters the water to chase or retrieve prey underneath the water’s surface (Duffy 1983; Haney and Stone 1988). The estimated Laridae and Procellariidae pairwise divergence was 71 million years ago, and these families are not considered close relatives (Slack et al. 2006).

**Table 1:** Species studied and the number of individuals

Order	Family	Species	*N*
Charadriiformes	Laridae	*Anous stolidu*s Brown Noddy	3
		*Anous tenuirostris* Lesser Noddy	2
		*Gygis alba* White Tern	2
		*Onychoprion fuscatus* Sooty Tern	3
		*Larosterna inc*a Inca Tern	3
Procellariiformes	Procellariidae	*Ardenna pacifica* Wedge-tailed Shearwater	2
		*Puffinus bailloni* Tropical Shearwater	3

Inca Terns were studied at Birdworld, Farnham, Surrey, UK (51.813, − 0.840°) in May and July 2023, and the other species were studied at Aride Island Nature Reserve, Seychelles (− 4.214, 55.668°) in June 2023. All Inca Terns were adults that had been held in captivity for several years. Individual birds were caught in their holding enclosure using hand nets and placed in a cloth bag and carried to a nearby building where measurements were made. On completion of measurements, each bird was released immediately into its enclosure. Of the six species studied on Aride Island the Sooty Terns and Brown Noddies were caught using hand nets, Tropical Shearwaters, and Wedge-tailed Shearwaters were caught by hand, and White Terns and Lesser Noddies were caught with mist nets. All birds were adults. In both locations, measurements of visual fields were conducted in a darkened room, and on the completion of measurements, each bird was released within 65 min near to its capture location. Birds were walked from the site of capture to the darkened room for a maximum of 10 min.

### Visual field measurements

**Fig. 1:**
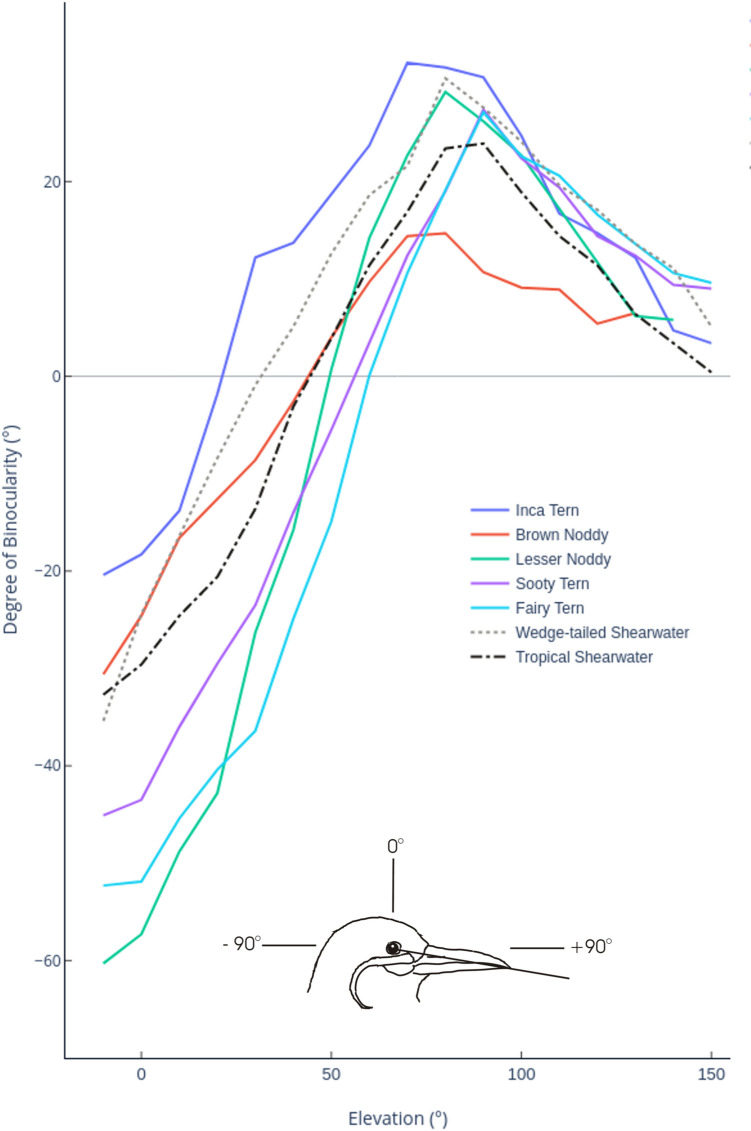
The mean angular separation of the retinal field margins in the anterior portion of the visual field as a function of elevation in the median sagittal plane of the head. Positive values indicate the width of the binocular field, negative values indicate the width of the blind area. The coordinate system is such that the horizontal plane is defined by the elevations − 90° (behind the head) and + 90° (in front of the head), and 0° is directly above the head. The drawing shows a bird’s head in profile with key coordinates indicated and the visual projection of the eye–bill tip axis. The head position shown is approximately that spontaneously adopted by an Inca Tern when held in the hand and indicates the head position at which visual field parameters were measured. The species studied were; *Anous stolidus* (Brown Noddies)*, Anous tenuirostris* (Lesser Noddies), *Gygis alba* (White Terns), *Onychoprion fuscatus* (Sooty Terns), *Larosterna inca* (Inca Terns), *Ardenna pacifica* (Wedge-tailed Shearwaters), and *Puffinus bailloni* (Tropical Shearwaters). An alternate black and white version of Fig. 1 can be found in the supplementary information

**Fig. 2:**
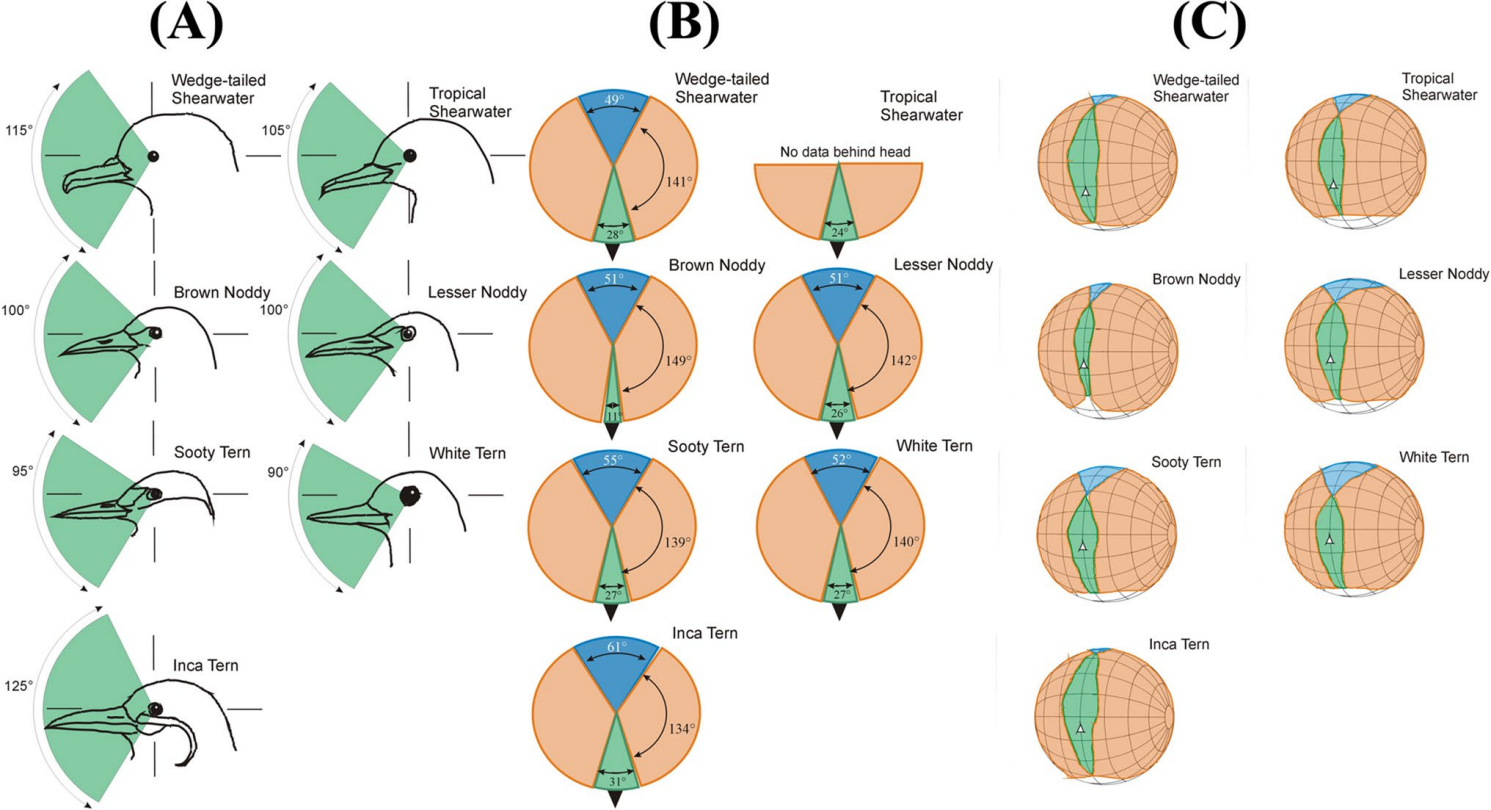
Visual fields of seven tropical seabird species. **A Left panel** vertical sections of the binocular field of each species in the median sagittal plane of the head. **B Middle panel** horizontal sections of the visual field of each species, the position of the horizontal plane is indicated by the horizontal lines shown in the vertical sections and in the schematic head and co-ordinate diagram shown in Fig. 1. **C Right panel** orthographic projection of the visual fields of each species. These figures use the conventional latitude and longitude coordinate system where the equator is vertically aligned with the head’s median sagittal plane, the grid is at 20° intervals. It should be imagined that the birds’ head is placed at the centre of the sphere with the visual field regions projected outwards onto the sphere’s surface. Colours are used to indicate monocular (orange), the binocular (green), and blind (blue) portions of the visual fields. The white and black triangles indicates the directions of the eye bill-tip projections. See Table 1 for species Latin names

The ophthalmoscopic reflex technique was used to measure the visual field characteristics, following the standard procedure described in previous studies (Martin et al. 2007; Martin and Wanless 2015; Cantlay et al. 2020). Each bird was held with its body immobilised in a foam rubber cradle and its bill placed in a holder specially designed for each species, with the head of the bird adopting its natural resting position. This arrangement fixed the at-rest head position with respect to the co-ordinate system used to characterise the visual field (Martin et al. 2007). This technique has been consistently applied across a wide taxonomy of avian species and provides a reliable method for interspecific comparisons of visual field topography (Martin 2017). The UK Animals (Scientific Procedures) Act 1986 is not applicable due to the procedure being non-invasive and the short period of time (generally 30 min) for bird restraint (Martin and Portugal 2011). Spontaneous eye movements were observed in some of the species. These refer to the observation that some bird species have complex rotational eye movements, and the translational effect of these movements can alter the limits of the visual field recorded at each elevation (Martin et al. 2008). Visual field measurements were taken for the positions that the eyes spontaneously adopted when fully rotated forward, hence converged for the front field, and provides an estimate of maximum binocular field width (Potier et al. 2018). In each species, detailed measurements were made only throughout the anterior portion of the visual field with a single set of measurements made at − 90° (directly behind the head; see Fig. 1 for the explanation of co-ordinate system) to determine the width of the blind area and thus allow characterisation of the monocular fields in the horizontal plane. Mean visual field data for each species were determined and used to create vertical (Fig. 2a, left panel) and horizontal (Fig. 2b, centre panel) sections through the visual fields and topographical maps of the anterior fields (Fig. 2c, right panel) for each species.

The small number of species measured in this study precluded a full phylogenetic generalised least squares analysis of the kind employed in the investigation of the relationship between visual fields and foraging in Anatidae (Cantlay, et al. 2023) and Strigidae (Potier et al. 2023), and thus our results are descriptive.

## Results

### General features of the visual fields

The general topography of the binocular visual field of all seven species is relatively similar (Figs. 1 and 2). All species have a vertically long and narrow binocular field, with the visual projection of the eye bill-tip occurring within the ventral portion of the binocular field below the point of its maximum width. All species have a blind area which starts in the dorsal quadrant of the frontal field and extends above and behind the head down to the horizontal.

### Interspecific differences in visual fields

Within these shared general features of the visual fields, there are clear interspecific differences: (1) the binocular region, from the elevation at which it could be measured below the bill, extends vertically through between 90° (White Terns) and 125° (Inca Terns), with a mean vertical extent of 104° (mean of all species), (2) the maximum width of the binocular fields differed between 15° (Brown Noddy) and 32° (Inca Tern) and the mean maximum width across all species equalled 26°, (3) the maximum width of the binocular region projects above the horizontal and lies at a mean value of 17° above the direction of the eye bill-tip projections, (4) laterally, there are extensive regions on monocular vision ranging from 134° (Inca Tern) to 149° (Brown Noddy) with a mean width across all species of 140°, and (5) in all species there is a blind region that projects into the dorsal anterior sector of the visual field, it starts at an elevation of 55° in Fairy Terns and 25° in Inca Terns and has a mean width across all species of approximately 35° directly above the head, (6) the width of the blind sector directly behind the head (elevation − 90°) varies between 49° (Wedge-tailed Shearwater) and 61° (Inca Tern) with a mean width across all species of approximately 53°.

## Discussion

### General characteristics of the visual fields

The visual fields of the seven seabird species show the characteristics of birds which rely on visual cues to guide the accurate placement and accurate timing of arrival of their bill at a target (Martin 2009). These visual field characteristics are driven particularly by the visual tasks associated with foraging for discrete targets, as opposed to using tactile cues or filter-feeding techniques. The key visual field characteristics that underlie this interpretation of these birds’ visual field characteristics are the vertically long and relatively narrow binocular field within which the visual projection of the bill tip is positioned below the region of maximum binocular field width (Fig. 2). It has been argued (Martin 2009) that this configuration serves to provide optic flow-field information that is necessary for both accurate placement of the bill with respect to a target and for accurate estimation of time to contact the target, which is necessary for precise timing of bill opening when seizing prey, along with playing a pivotal role in obstacle avoidance (Bhagavatula et al. 2011). These kinds of visually guided tasks are practiced by all species in the present sample regardless of whether they forage using dipping for items at the sea surface or plunging for items taken at or below the sea surface. Previous studies on Procellarids documented binocular field widths of 30°, 27°, and 32° for White-chinned Petrels (*Procellaria aequinoctialis*), Grey-headed (*Diomedia chrysostoma*) and Black-browed Albatrosses (*D. melanophris*), respectively (Martin 1998; Martin and Prince 2001), compared to the 24° and 28° binocular width in Tropical and Wedge-tailed Shearwaters found in the present study.

This similarity in visual field configuration between the Laridae and Procellariidae species in this sample is noteworthy because these taxa are not considered to be closely related, their lineages having diverged about 71 million mya (Slack et al. 2006). It reinforces the idea that, in birds in general, visual field characteristics are driven primarily by the visual demands of foraging rather than phylogeny. This accords with similar conclusions drawn from studies of visual fields of a larger sample of species using phylogenetic generalised least squares analysis in ducks, geese, and swans (Anseriformes) (Cantlay et al. 2023) and in owls (Strigiformes) (Potier et al. 2023).

### Interspecific differences in visual fields

While there is a similarity in the overall characteristics of the visual fields of this sample of seabirds, there are also clear differences in the dimensions of specific aspects of their visual fields. These include differences in the maximum width and vertical extent of binocular fields, and positions of the binocular field relative to the eye bill-tip projection. These differences are relatively subtle compared with the wide range of visual field configurations recorded in birds (Martin 2017). However, it has been shown that relatively small differences in visual field topography among closely related birds can be accounted for by consideration of differences in foraging behaviour and diet, and the nature of the associated visual challenges of different foraging task. This has been shown in comparisons between visual fields and foraging behaviour among species of ibis (Threskiornithidae) (Martin and Portugal 2011), ducks (Anatidae) (Guillemain et al. 2002; Martin et al. 2007), auks (Alcidae) (Martin and Wanless 2015), plovers (Charadriidae) (Martin and Piersma 2009; Cantlay et al. 2019), vultures and hawks (Accipitridae) (Portugal et al. 2017; 2023), and petrels (Procellariidae) (Martin and Prince 2001).

These studies suggest that visual fields can be fine-tuned to specific aspects of the foraging ecology of species within the broad parameters required for successful visually guided foraging. The particular ways in which the interspecific differences in the visual fields of the shearwaters and terns described here can be interpreted with respect to different foraging tasks require a detailed analysis of these species’ foraging ecology and behaviours. For example, differences between the visual fields of Inca and Noddy Terns are likely to result from differences in the tasks of detecting and acquiring their preferred prey types. Brown Noddies are described as feeding, “mainly by hover-dipping and contact-dipping; regularly food patters at surface… usually does not plunge-dive. Captures flying-fish in air… Forages on moonlit nights” (Gochfeld and Burger 1996). Inca Terns are described as feeding “mainly on small anchoveta (*Engraulis ringens*), also planktonic crustaceans; offal and scraps… Forages mainly by plunge-diving, and contact – and surface-dipping; scavenges for scraps left by sea lions and avian predators” (Gochfeld and Burger 1996). Thus, Noddy Terns feed mainly in the air, but they have the exacting perceptual demand associated with taking flying prey (flying fish, Exocoetidae**)** whose appearance is intermittent and brief. On the other hand, Inca Terns feed below the water surface on small but less evasive prey (planktonic crustaceans) (Duffy 1983; Hanley and Stone 1988; Gochfeld and Burger 1996). This means that although both species have to achieve accurate location and timing of prey capture for successful foraging their tasks are quite different and how these different tasks will have driven the fine-tuning and difference between their visual fields would require further detailed analysis of their foraging tasks. Amphibious vision has particular demands compared to foraging in the air due to the loss of corneal power and the narrowing of the visual fields on entering the water (Katzir and Howland 2003), and the latter may be sufficient to account for the broader binocular field in Inca Terns compared with those in Brown Noddy (Fig. 2). To be certain of this requires more detailed knowledge of the optical structure of the eyes in these species, but entering water can result in the reduction of maximum binocular field width by approximately 50% (Martin and Young 1984). Thus, it would be predicted that upon immersion the maximum binocular field of Inca Terns (34°) would narrow to about 17°, which is similar to the maximum width of the Brown Noddy 15° in air, with the results that the binocular fields of both species are of similar width when the birds are engaged in their primary foraging tasks. This is consistent with the fact that the three species that forage using dive-plunging have the longest vertical extension of binocular vision (Inca terns, 125°; Wedge-tailed shearwaters, 115°; Tropical shearwaters, 100°) compared to those that do not dive. The two species (Lesser Noddy, Inca Tern) not reported to forage at night or crepuscular hours do not appear to show any particularity in their visual fields. Clearly, a more detailed analysis of the foraging ecology and their associated perceptual challenges across all of the species (e.g., Regular et al. 2011) are required to determine factors which may have led to the recorded differences in their visual fields.

### Supplementary Information

Below is the link to the electronic supplementary material.

Supplementary file1 (DOCX 87 KB)

## Data Availability

All data are available in the Supplementary information.
