# Supplementary material for: A seabird’s eye view: visual fields of some seabirds (Laridae and Procellariidae) from tropical latitudes
Source: Naturwissenschaften. 2024 Jul 17;111(4):40. doi: 10.1007/s00114-024-01926-4 (PMC11254976; doi:10.1007/s00114-024-01926-4)
Supplement: Supplementary file 1 — Supplementary file1 (DOCX 87 KB) [file 114_2024_1926_MOESM1_ESM.docx]

**Supplementary Information for:**

**A seabird’s eye view: the visual fields of tropical seabirds**

**Eleanor A. Lucas¹^*^, Graham R. Martin^2^, Gérard Rocamora^3,4^ and Steven J. Portugal¹^,5,6^**

^1^*Department of Biological Sciences, School of Life and Environmental Sciences, Royal Holloway, University of London, Egham, Surrey, TW20 0EX, UK*

*^2^School of Biosciences, University of Birmingham, Edgbaston, Birmingham, B15 2TT, UK*

*^3^Island Conservation Society, Mahé, Seychelles*

*^4^Island Biodiversity and Conservation centre, University of Seychelles, Mahé, Seychelles*

*^5^The Natural History Museum Tring, Akeman Street, Tring, Herts, HP23 6AP, UK*

*^6^Department of Biology, University of Oxford, Oxford, OX1 3SZ, UK*

**Supplementary Table S1.** Full table of mean maximum binocular field widths for all seven species, across perimeter elevations of -10**°** to 150°. Blank spaces within the table indicate areas about the head of each bird which were not accessible, therefore data could not be collected.

| **Elevation**  **(°)** | **Inca Tern** | **Brown Noddy** | **Lesser Noddy** | **Tropical Shearwater** | **Sooty Tern** | **White Tern** | **Wedge-Tailed Shearwater** |
| --- | --- | --- | --- | --- | --- | --- | --- |
| **-10** |  | -30.6 | -60.3 |  |  |  | -35.4 |
| **0** | -18.3 | -24.6 | -57.3 | -29.6 | -43.5 | -51.9 | -24.4 |
| **10** | -13.8 | -16.6 | -48.8 | -24.6 | -36 | -45.4 | -16.4 |
| **20** | -1.8 | -12.6 | -42.8 | -20.6 | -29.5 | -40.4 | -8.4 |
| **30** | 12.2 | -8.6 | -26.3 | -13.6 | -23.5 | -36.4 | -0.9 |
| **40** | 13.7 | -2.6 | -15.8 | -3.1 | -14 | -24.9 | 5.1 |
| **50** | 18.7 | 4 | 0.7 | 3.9 | -5.5 | -14.9 | 12.6 |
| **60** | 23.7 | 9.7 | 14.2 | 11.4 | 3.4 | 0.1 | 18.6 |
| **70** | 32.2 | 14.4 | 22.7 | 16.9 | 12.4 | 10.6 | 21.6 |
| **80** | 31.7 | 14.7 | 29.2 | 23.4 | 19 | 19.1 | 30.6 |
| **90** | 30.7 | 10.7 | 26.2 | 23.9 | 27.4 | 27.1 | 27.6 |
| **100** | 24.7 | 9.1 | 22.7 | 18.9 | 22.4 | 22.6 | 24.1 |
| **110** | 16.7 | 8.9 | 17.2 | 14.4 | 19.4 | 20.6 | 19.6 |
| **120** | 14.7 | 5.4 | 11.7 | 11.4 | 14.4 | 16.6 | 17.1 |
| **130** | 12.2 |  | 6.2 | 6.4 | 12.4 | 13.6 | 13.6 |
| **140** | 4.7 |  |  | 3.4 | 9.4 | 10.6 | 11.1 |
| **150** |  |  |  | 0.4 |  |  | 5.1 |

**Supplementary Table S2.** Data for each of the seven seabird species, including data for the four binocular field parameters; maximum binocular field width, binocular onset, blind spot at 90 degrees, and binocularity before 90 degrees. Also includes life history data for each species; body mass, foraging method, primary diet, nocturnal habits and locomotory foraging niche. Information on diet, nocturnal feeding and other foraging aspects have been obtained from: Carboneras, 1992; Gochfeld & Burger, 1996; Catry, Ramos, Jaquemet et al., 2009; Catry, Ramos, Le Corre et al., 2009; Feare, 2013; Skerrett, 2013; Wilman et al., 2014; Calabrese, 2015; Ravache et al., 2020).

| **Species** | **Latin name** | **Max bin** | **Bin onset** | **Blind area width at 90** | **Binoc before 90** | **Mass** | **Foraging techniques** | **Primary Diet =** | **Nocturnal foraging** | **Locomotory foraging niche** |
| --- | --- | --- | --- | --- | --- | --- | --- | --- | --- | --- |
| Inca Tern | *Larosterna inca* | 32.2 | 30 | 59.3 | 60 | 219 | dipping and plunging (shallow dives) | Mainly small fish, also crustaceans | no | aerial |
| Brown Noddy | *Anous stolidus* | 14.7 | 70 | 49.6 | 20 | 177.8 | dipping (surface feeder) | Mainly small fish, some squid | yes | aerial |
| Lesser Noddy | *Anous tenuirostris* | 29.2 | 50 | 50.3 | 40 | 112 | dipping (surface feeder) | Small fish | no | aerial |
| Tropical Shearwater | *Puffinus*  *bailloni* | 23.9 | 50 | 58.6 | 40 | 192.8 | plunging  (deep dives) and dipping) | mainly small fish; some squid | yes | aquatic/  aerial |
| Sooty Tern | *Onychoprion fuscatus* | 27.4 | 60 | 63.5 | 30 | 185.7 | dipping (surface feeder) | Mainly fish and squid | yes | aerial |
| White Tern | *Gygis alba* | 27.1 | 60 | 53.4 | 30 | 117 | dipping  (surface feeder) | mainly small fish; some squid | yes | aerial |
| Wedge-Tailed Shearwater | *Ardenna pacifica* | 30.6 | 40 | 47.4 | 50 | 388 | Dipping and plunging  (shallow dives) | mainly fish; also squid | yes | aquatic/  aerial |


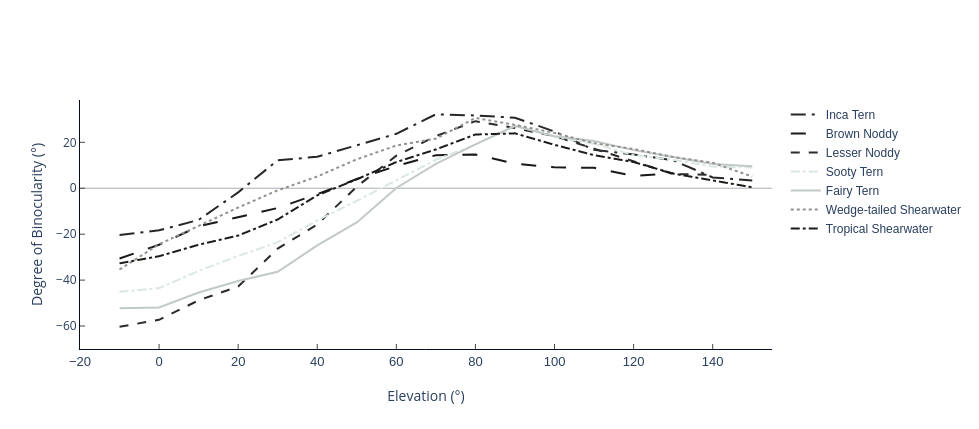


**Supplementary Figure 1.** The mean angular separation of the retinal field margins in the anterior portion of the visual field as a function of elevation in the median sagittal plane of the head. Positive values indicate the width of the binocular field, negative values indicate the width of the blind area. The coordinate system is such that the horizontal plane is defined by the elevations - 90° (behind the head) and + 90° (in front of the head), and 0° is directly above the head. The drawing shows a bird’s head in profile with key coordinates indicated and the visual projection of the eye–bill tip axis. The head position shown is approximately that spontaneously adopted by an Inca Tern when held in the hand and indicates the head position at which visual field parameters were measured. The species studied were; Anous stolidus (Brown Noddies), Anous tenuirostris (Lesser Noddies), Gygis alba (White Terns), Onychoprion fuscatus (Sooty Terns), Larosterna inca (Inca Terns), Ardenna pacifica (Wedge-tailed Shearwaters) and Puffinus bailloni (Tropical Shearwaters). An alternate black and white version of Figure 1 can be found in the supplementary information.

**Supplementary References**

Calabrese L. (2015). *Foraging ecology and breeding biology of Wedge-­tailed shearwater (Puffinus pacificus) and Tropical shearwater (Puffinus bailloni) on Aride Island Nature Reserve, Seychelles: tools for conservation*. Centre d’Etudes Biologiques de Chizé (CNRS) – Island Conservation Society (Seychelles) – Université Pierre et Marie Curie, Paris, 132 p.

Carboneras, C. (1992). Family Procellariidae (Petrels and Shearwaters). Pp. 216-257 in del Hoyo, J., Elliott A. and Sargatal J. Eds. *Handbook of the Birds of the World*. *Vol 1. Ostrich to Dutch.* Lynx Edicions, Barcelona.

Catry, T., Ramos, J. A., Jaquemet, S., Faulquier, L., Berlincourt, M., Hauselmann, A., Pinet, P., & Le Corre, M. (2009). Comparative foraging ecology of a tropical seabird community of the Seychelles, western Indian Ocean. *Marine Ecology Progress Series*, *374*, 259–272.

Catry, T., Ramos, J. A., Le Corre, M., & Phillips, R. A. (2009). Movements, at-sea distribution and behaviour of a tropical pelagic seabird: the wedge-tailed shearwater in the western Indian Ocean. *Marine Ecology Progress Series*, *391*, 231–242.

Feare, A. (2013). Sooty tern *Onychoprion fuscatus*. Pp. 462-467 in Safford, R. J. and Hawkins, A. F. A. (eds). *The Birds of Africa. Volume VIII: The Malagasy Region*. London: Christopher Helm.

Gochfeld, M. and Burger, J. (1996). Family Sternidae (Terns) Pp. 624-667 in del Hoyo, J., Elliott, A. and Sargatal, J. eds. Handbook of the Birds of the World Vol. 3. Lynx Edicions, Barcelona.

Ravache, A., Bourgeois, K., Thibault, M., Dromzée, S., Weimerskirch, H., de Grissac, S., Prudor, A., Lorrain, A., Menkes, C., & Allain, V. (2020). Flying to the moon: Lunar cycle influences trip duration and nocturnal foraging behavior of the wedge-tailed shearwater *Ardenna pacifica*. *Journal of Experimental Marine Biology and Ecology*, *525*, 151322.

Skerrett, A. (2013). Brown noddy *Anous stolidus*; Lesser noddy *Anous tenuirostris;* White-tern *Gygis alba*. Pp. 470-477 in Safford, R. J. and Hawkins, A. F. A. (eds). *The Birds of Africa. Volume VIII: The Malagasy Region*. London: Christopher Helm.

Wilman, H., Belmaker, J., Simpson, J., de la Rosa, C., Rivadeneira, M. M., & Jetz, W. (2014). EltonTraits 1.0: Species‐level foraging attributes of the world’s birds and mammals: Ecological Archives E095‐178. *Ecology*, *95*(7), 2027.
